# Lizard polymorphic throat colors are distinguishable by conspecifics and predators across variable light environments

**DOI:** 10.1371/journal.pone.0334557

**Published:** 2025-10-16

**Authors:** Graham T. BeVier, Kinsey M. Brock

**Affiliations:** 1 Department of Biology, University of Nevada, Reno, Nevada, United States of America; 2 Department of Biology, College of Sciences, San Diego State University, San Diego, California, United States of America; 3 Biodiversity Museum, Department of Biology, College of Sciences, San Diego State University, San Diego, California, United States of America; HUN-REN Centre for Ecological Research, HUNGARY

## Abstract

Color polymorphisms, or distinct color variants within a population, provide tractable study systems for studying the generation, maintenance, and loss of phenotypic diversity in nature because biologists can easily observe changes in the number and frequency of discrete variants over time. However, many color polymorphisms are studied in the context of the human visual system and do not consider how conspecifics or potential predators view morph variation. The visual systems of predators and conspecifics may be sensitive to different aspects of coloration, which can influence the evolution and maintenance of morph diversity and phenotypic variation within and between populations. The Aegean wall lizard (*Podarcis erhardii*) is a color polymorphic lizard that exhibits co-occurring orange, white, and yellow throat color morphs. Here, we measured the reflectance of *P. erhardii* throat color patches and used visual modelling to determine if lizards and their bird and snake predators can visually discriminate between morph colors across different lighting contexts. Our results suggest that *P*. *erhardii* and their violet-sensitive bird and snake predators can distinguish chromatically between each color morph pair in standard daylight and forest shade illuminance contexts. However, only *P. erhardii* can distinguish achromatic morph colors in both illuminance contexts (except for white and yellow morphs in forest shade). These results indicate that *P. erhardii* morphs are most difficult for predators to distinguish in low lighting conditions and could help explain previously observed morph differences in microhabitat usage.

## Introduction

Within and among species, animals employ many types of colorful signals to broadcast information [1–4]. Color polymorphism can operate as one such color signal, where the presence of two or more genetically-determined color morphs exist within a breeding population, with the rarer morph persisting due to a mechanism other than recurrent mutation [5,6]. Color polymorphism is common, having evolved in every major animal group in the tree of life [6–8]. Lizards have evolved strikingly similar ventral color polymorphisms in phylogenetically distant families such as the Agamidae [9], Lacertidae [10], and Phrynosomatidae [11,12]. For the coloration of alternative morphs to function as social signals, conspecifics must be able to perceive that variation in conspecific color as categorically discriminable [9,13,14], which has not yet been confirmed for many lizard species.

Determining how distinguishable color morphs are to conspecifics and predators can help us understand the social, ecological, and environmental factors that generate, maintain, and erode morph diversity within and among populations. Although a morph with a bold visual signal may attract more mates [15,16] or communicate fighting ability and ward off would-be conspecific combatants [17–19], it could also draw increased attention from predators [20–22]. As such, color signals represent a balance struck between two opposing selection pressures: the benefit of conveying information to conspecifics against the risk of increased detection by visual predators. Because the visual sensitivities of many animals, including lizards and their predators, are known to differ from those of humans [23,24], it is critical to assess how morph coloration appears to the specific visual systems of the species in question and the animals searching them out for a meal.

Color polymorphic lacertid lizards provide an excellent system for the study of visual ecology because many closely related species share a ventral orange, yellow, and white polymorphism [10]. Previous research characterizing the spectral sensitivities of two lacertid species (*Podarcis muralis* and *Zootoca vivipara*) found that their visual systems are comprised of pure-cone retinas with one spectral class of double cones and four spectral classes of single cones [25,26]. The four types of single cones result in color vision sensitive to wavelengths ranging from 320 to 700 nm, whereas the long-wavelength double cones are theorized to serve achromatic perception [27–29]. Lizard color sensitivities differ from those of their main predators such as birds and snakes. Raptors are sensitive to wavelengths from 400 to 700 nm and filter out ultraviolet (UV) cues for detection [30]. Snakes display substantial diversity in ocular anatomy, which are highly divergent as compared to other squamates [31]. Indeed, eye transmittance, visual pigment tuning, and retinal anatomy all vary considerably among snake taxa. As such, the relative detectability of alternative morph colors is dependent upon disparate variables which likely differ between conspecific and predator observers. Differences in morph detection by predators could have crucial consequences for lizards if it results in different predation levels. Previous research on intraspecific color morphs has found that predators develop preferences for certain morphs over others [32–34], which may exert an additional level of selection on morphs beyond discriminability of coloration by predator visual systems.

**Fig 1:**
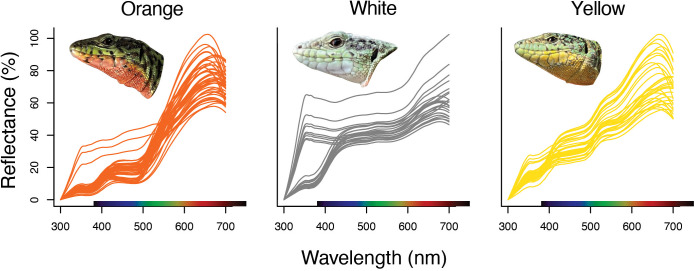
*P. erhardii* monochromatic throat color morph reflectance spectra. Each line represents the mean wavelength for each individual used in this study. The ultraviolet spectrum ranges from 300-400 nm. The visible spectrum ranges from 400-700 nm.

The Aegean wall lizard (*Podarcis erhardii*) is a color polymorphic lacertid lizard endemic to the southern Balkans and hundreds of Aegean Islands [35,36]. *Podarcis erhardii* is just one of 28 color polymorphic wall lizards in the genus *Podarcis* [10] whose throat coloration can be monochromatic (white, yellow, or orange) or mosaic (white-yellow, white-orange, or yellow-orange) (Fig 1; [10,37]). Polymorphic color patches in this species are restricted to the throat [37], which can be selectively used to broadcast morph identity to conspecifics, while simultaneously reducing visibility to predators. Indeed, *Podarcis erhardii* performs conspecific signalling behaviors that expose the color polymorphic throat region to visual predators, primarily through throat displays that involve tilting the head back to present the throat [38]. Additionally, *P. erhardii* frequently climbs walls vertically and basks in this position that would expose the throat to any conspecifics or predators above, such as birds. Polymorphic throat coloration develops at sexual maturity in all sexes and remains unchanged like in other *Podarcis* species [39,40]. Polymorphism in *Podarcis* species seems to be genetically-determined, and genetic differences between morphs are restricted to small genome regions attendant to pterin and carotenoid metabolism [40]. Within and between populations of color polymorphic *Podarcis*, the frequency of color morphs varies considerably [41,42]. *P. erhardii* is a habitat generalist and can be found anywhere from sea-level sand dunes to montane open forests but seems to thrive in rocky areas with low, spiny phrygana vegetation and grasses and dry stone walls [43]. Microhabitat usage differs between color morphs, where orange morphs tend to utilize shaded areas in vegetative cover and low-light microhabitat more often than white and yellow morphs [44]. *Podarcis erhardii* is predated upon by numerous raptor (e.g., *Buteo buteo*, *Falco tinnunculus*), corvid (*Corvus* spp.), and snake species (e.g., *Elaphe quatuorlineata*, *Eryx jaculus, Natrix natrix*, *Hierophis gemonensis, Vipera ammodytes*) [45]. These predation pressures differ between habitat contexts (i.e., small islet vs. mainland) [46,47], which has ecological ramifications for color morph maintenance throughout its island range [42].

In this study we determined if *P. erhardii’*s polymorphic throat colors are distinguishable by both conspecifics and two of the most common predators of this species, birds and snakes. We used visual modelling of spectral data to determine if *P. erhardii* throat color patches can be distinguished by the visual systems of conspecifics and predators across variable lighting conditions that represent the diverse microhabitat conditions this species is known to occupy. Across these different lighting environments, the availability of certain wavelengths in the visible light range is differentially restricted and thus alter how the coloration of *P. erhardii* morphs is perceived by conspecifics and predators alike. Herein, we address the following questions: (1) can *P. erhardii* visually discriminate between conspecific color morphs, (2) are birds and snakes able to visually discriminate between these color morphs, and (3) does color discrimination by *P. erhardii,* birds and snakes differ between different lighting contexts? We predicted that the *P. erhardii* visual system would be able to differentiate between the three throat color morphs better than the bird and snake visual systems, and that they would be able to do so under two illuminance contexts: standard daylight and forest shade conditions, probably due to strong selection for discerning morph types in social situations [38].

## Materials and methods

In June 2017, we sampled lizards (N = 102; 52M:50F) from a site near the village of Moni (elev. 590 m a.s.l.; 37.08043203432896 ºN, 25.49171180549804 ºE) on the island of Naxos, Greece. We chose this site for its high density of polymorphic wall lizards and diverse lighting environments. Measuring mosaic morph coloration presents methodological challenges like small or irregular scale shapes at landmark scale locations; therefore, mosaic morph lizards were excluded from this study. We caught the monochromatic study animals using a 2.7 m telescopic fishing pole with a thread lasso at the end. Upon capture, we measured the snout-vent length (mm) of the lizards using Mitutoyo digital precision calipers (Mitutoyo America Corporation, Aurora, Illinois, USA). Lizards smaller than 45 mm were deemed unfit for color and morphometric measurements and immediately returned to the spot of capture [37].

To quantify the throat color of our specimens, we first used an Ocean Optics Flame S-UV-VIS Fiber Optic Spectrometer 200−850 nm (Ocean Optics Inc., Dunedin, FL, USA) and Xenon pulse light source connected to a probe with a fiber optic cable. We calibrated these measurements using a white WS-1-SL Labsphere Diffuse Reflectance Standard (Spectralon, Ocean Optics Inc.). To collect spectral data, we placed the spectrometer illumination probe 5 mm away from the skin, perpendicular to the surface of the throat [37,39,48]. We collected six spectral measurements for each individual at landmark throat scale locations, which consisted of a sample point measuring 3 mm in diameter. In this way, we minimized potential measurement bias and characterized variation across the entire throat signal.

Spectral data were imported and analyzed in R version 2023.06.2 + 561 [49], principally using the R package ‘pavo’ [50,51]. We ensured these spectral data ranged from 300 to 700 nm, then averaged the six spectral measurements catalogued with each individual lizard using the ‘aggspec’ function, producing one measurement for subsequent analyses. After confirming no sub-zero values existed in the data, we smoothed spectra with an optimal span of 0.2 using ‘procspec’ to reduce electrical noise while preserving the shape of the curves [9,50]. We then retrieved common colorimetric variables of mean brightness (mean relative reflectance over the entire spectral range), chroma ((R_max_ - R_min_)/mean brightness), and hue (wavelength at middle reflectance), for each morph and sex to use as inputs in visual models [52,53]. All data used in this study are available in the Supporting Information as a.zip file.

We created visual models that correspond with lizard visual systems and those of two predators: birds and colubrid snakes [26,47,54]. In *Podarcis muralis*, research suggests that four-cone visual systems are likely, but we do not yet have direct evidence confirming this hypothesis [25]. Given this, for our *P. erhardii* visual model we constructed a custom tetrachromat model using the ‘sensmodel’ function, using the peak cone sensitivities described for *P. muralis* [26] since visual systems are phylogenetically conserved within lizard families [2]. Here, we assumed a relative cone abundance ratio of 1:1:1:4, which corresponds to UV-wavelength, short-wavelength (SW), medium-wavelength (MW), and long-wavelength (LW) sensitive cones, respectively [14,26]. For our bird visual model, we built a custom tetrachromat model based on cone sensitivities of common buzzards (*Buteo buteo*; [30]), assuming a relative cone abundance ratio of 1:2:2:4 [55]. Throughout its range, *P. erhardii* is predated upon by corvids and raptors such as *B. buteo* [45,56], who have the same VS class of avian color vision shared by *Pavo cristatus* [57,58]; as such, this model has been used as an analog for the visual systems of common raptor predators of *P. erhardii* in previous studies [59]. We based our snake model on the retinal profile and cone class ratios of the garter snake (*Thamnophis sirtalis*; [54]). *T. sirtalis* is a diurnal, visually hunting snake that shares a common ancestor and spectral sensitivities with grass and rat snakes (*Natrix natrix* and *Elaphe quatuorlineata*; [31]), colubrid predators of *P. erhardii* [45]. For both the lizard and snake visual models, we adopted a Weber fraction of 0.05 for the LW-sensitive cone [14,26,60,61], whereas we used 0.10 for the avian model [30].

We altered the illuminance assumptions of all three taxonomic visual models to include ‘d65’ and ‘forestshade’ conditions, resulting in six total visual models. These assumptions describe ambient light environments, where the availability of certain wavelengths is differentially restricted across the wavelength range. The illuminant ‘d65’ refers to the CIE Standard Illuminant D65 [62], which represents standard daylight and has a relative spectral power distribution that peaks at 460 nm. The illuminant ‘forest shade’ characterizes the light conditions of forest shade as “yellow-green” among global localities, depicting a common peak around 550 nm with a sharp increase around 680 nm [63]. *P. erhardii*, birds and snakes alike utilize both habitat types.

We tested whether the three *P. erhardii* throat color morphs are distinguishable by wall lizard, raptor, and colubrid snake visual systems in each of the two illuminance conditions of interest. To do so, we utilized a distance-based PERMANOVA on both the chromatic and achromatic contrasts generated by each of the six visual models. Chromatic and achromatic contrasts were calculated with the ‘coldist’ function, which returns the Euclidean distance in colorspace between two points to quantify the contrast between them [55]. Specifically, we created distance matrices for both chromatic and achromatic contrasts under each illuminance condition for the three visual systems, then used the ‘pairwise.adonis’ function from the ‘pairwiseAdonis’ R package [64] to make multi-level pairwise comparisons between morph groups. We tested the assumption of multivariate homogeneity of group dispersions (variances) with the function ‘betadisper’ from the ‘vegan’ R package, which performs a multivariate analogue of Levene’s test. We found that the chromatic and achromatic contrasts for wall lizard, raptor, and snake visual systems alike were unequal for the two lighting contexts. However, because our largest morph group had the highest variance in each testing instance, our distance-based PERMANOVA procedure was not appreciably impacted by this heterogeneity and did not require any transformations to the data [65]. For these pairwise comparisons, we generated 999 permutations for the null, recording statistical significance (α = 0.05), a pseudo *F* statistic, and *R*^2^ as an estimate of effect size. PERMANOVAs were performed separately for each visual system and illuminance condition combination, resulting in six separate test outputs for both chromatic and achromatic contrasts.

We used the receptor noise model [55] to estimate the perceptual distance between colored stimuli and thus quantitatively discriminate between color morphs. Using the ‘bootcoldist’ function, we performed a bootstrap-based color distance comparison to generate confidence intervals for the mean chromatic and achromatic distances between each individual’s throat color average. We ran this procedure for each of the three visual systems under the two aforementioned illuminance conditions. This model returned chromatic and achromatic distances between the spectra of each morph pair (i.e., white-orange, white-yellow, yellow-orange) in terms of just noticeable differences (JND). 1 JND is described as the threshold of discrimination between two colors [60]; values <1 JND indicate the colors are indistinguishable, whereas for values >1 JND, the higher the value, the greater the distance in color space between the two colors. Pairs of colors that return values of 3 or more JND are easily discriminable, even in poor lighting conditions [14,60].

All research with live animals was conducted in accordance with the University of California, Merced Institutional Animal Care and Use Committee (IACUC protocol AUP17−0002) and permits provided by the Greek Ministry for Environment and Energy (Ψ4Γ64653Π8-ΗΛ5 assigned to K.M. Brock). No protected areas or species were sampled for this study.

## Results

**Fig 2:**
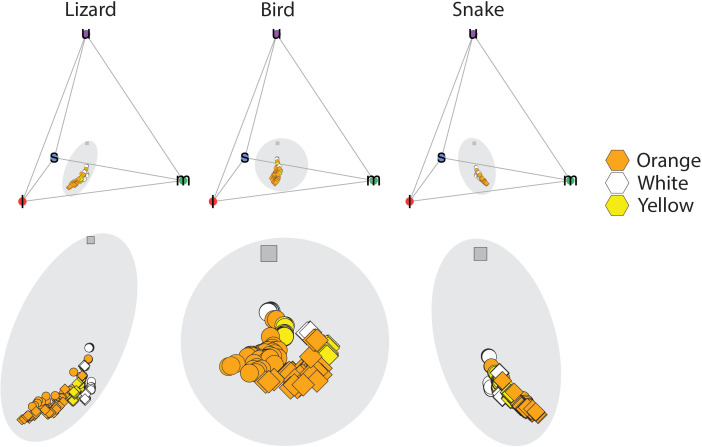
Distributions of *P. erhardii* color morphs in tetrahedral color space in the lizard (left), bird (middle), and snake (right) visual systems. Circle points represent d65 (standard daylight) illuminance and diamonds represent forest shade illuminance assumptions. The position of each point in the tetrahedron is determined by the relative stimulation of the four color cones (l = long-wavelength, m = medium-wavelength, s = short-wavelength, u = ultraviolet-wavelength) in visual systems of the Common wall lizard, *P. muralis* (left), a bird visual system, *B. buteo*, (middle), and a snake visual system, *Thamnophis sirtalis* (right). Each point is color-coded by morph (orange, white, and yellow) and the plot origins are indicated by dark grey squares.

**Fig 3:**
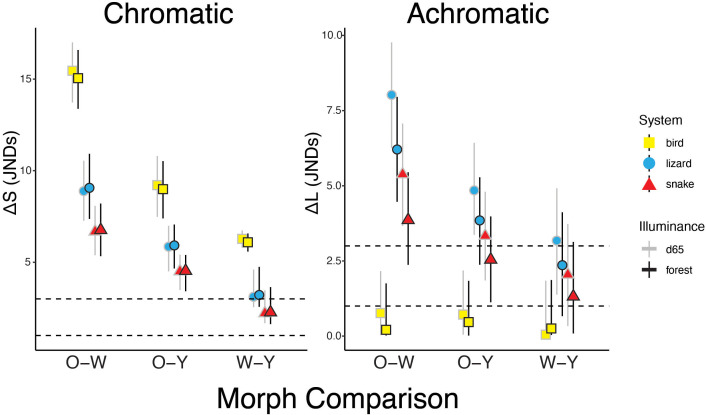
Chromatic (ΔS) and achromatic (ΔL) contrasts of *P. erhardii* color morphs through the eyes of birds, lizards, and snakes under different illuminance conditions. Means for each morph comparison are indicated with a circle (lizard visual system), square (bird visual system), and triangle (snake visual system). Confidence intervals (95%) are indicated by vertical lines. Horizontal dotted lines signify just noticeable difference (JND) cut-offs of 1 and 3, where points above the dotted lines indicate that the visual systems can perceive color differences. The illuminance condition for each morph comparison is indicated in greyscale and from left to right (d65 and forest).

Sampling details and colorimetric characteristics for each morph and sex group are reported in Table 1. In tetrahedral color space, the chromatic points of *P. erhardii* morph coloration are relatively segregated across illuminance conditions in lizard, raptor, and snake visual systems (Fig 2). Results from the receptor noise model procedure indicated that for lizard and raptor visual systems, *P. erhardii* color morph pairs are chromatically distinguishable in both illuminance contexts; however, morph pairs are not achromatically distinguishable by raptors (Fig 3). For snake visual systems, the mean chromatic distance between orange-white and orange-yellow pairs surpassed 3 JND in both illuminance contexts (Fig 3), whereas the white-yellow pair did not exceed this threshold in standard daylight or forest shade. The mean achromatic distance between morph pairs only surpassed 3 JND for the orange-white comparison in both illuminance conditions and the orange-yellow comparison in standard daylight (Fig 3).

**Table 1:** Colorimetric characteristics separated by morph and sex. Sample size (*n*), brightness (mean), chroma, and hue (λ at Rmid) are reported.

Morph (Sex)	*n*	Brightness	Chroma	Hue
Orange (M)	23	39.733	1.908635	543
Orange (F)	22	43.135	1.779565	536
White (M)	13	39.078	1.469239	415
White (F)	12	48.327	1.460344	409
Yellow (M)	16	43.484	1.615375	504
Yellow (F)	16	41.937	1.555434	500

Our distance-based PERMANOVA revealed that for lizard, avian, and snake visual models, the chromatic contrast between each color morph pair was statistically significant across the two illuminance conditions (Table 2). For lizards, the achromatic contrast between morph pairs was statistically significant for both lighting conditions except for the white-yellow comparison in forest shade (Table 3). For both raptors and colubrid snakes, achromatic contrast for the white-yellow comparison were not significant for either lighting condition, and the yellow-orange pair was not significant in forest shade (Table 3).

**Table 2:** Pairwise comparisons of color morph chromatic contrasts (∆S) for lizard, bird, and snake visual system models under two different illuminance conditions. Degrees of freedom (df), sum of squares (SS), pseudo *F* statistics, *R*^2^ as an estimate of effect size, and adjusted *P* values (Bonferroni corrections) are reported. Statistically significant results are bolded.

System	Illum	Comparison	df	SS	*F*	*P* (adj.)	*R* ^*2*^
Lizard	d65	white-yellow	1	0.5840196	19.17282	**<0.01**	0.2584885
		white-orange	1	1.7220814	48.04717	**<0.01**	0.4140314
		yellow-orange	1	2.3656576	88.00850	**<0.01**	0.5399013
	forest	white-yellow	1	0.5851351	18.90386	**<0.01**	0.2557899
		white-orange	1	1.7854895	49.25901	**<0.01**	0.4200872
		yellow-orange	1	2.4433677	91.28190	**<0.01**	0.5489587
Avian	d65	white-yellow	1	1.251889	163.3793	**<0.01**	0.7481446
		white-orange	1	3.578874	116.5468	**<0.01**	0.6315297
		yellow-orange	1	3.918981	137.7375	**<0.01**	0.6474529
	forest	white-yellow	1	1.257361	155.0318	**<0.01**	0.7381348
		white-orange	1	3.607919	117.9298	**<0.01**	0.634271
		yellow-orange	1	3.969749	141.2522	**<0.01**	0.653183
Snake	d65	white-yellow	1	0.487881	12.97840	**<0.01**	0.1909195
		white-orange	1	1.619141	40.20384	**<0.01**	0.3715565
		yellow-orange	1	2.297393	78.55529	**<0.01**	0.5115766
	forest	white-yellow	1	0.4801132	12.33032	**<0.01**	0.1831318
		white-orange	1	1.6834413	40.85103	**<0.01**	0.3752930
		yellow-orange	1	2.3787487	80.76467	**<0.01**	0.5185044

**Table 3:** Pairwise comparisons of color morph achromatic contrasts (∆L) for lizard, bird, and snake visual system models under two different illuminance conditions. Degrees of freedom (df), sum of squares (SS), pseudo *F* statistics, *R*^2^ as an estimate of effect size, and adjusted *P* values (Bonferroni corrections) are reported. Statistically significant results are bolded.

System	Illum	Comparison	df	SS	*F*	*P* (adj.)	*r* ^*2*^
Lizard	d65	white-yellow	1	0.3442611	5.787335	**0.015**	0.0952062
		white-orange	1	2.1522254	41.465636	**<0.01**	0.3788004
		yellow-orange	1	1.1391635	19.766490	**<0.01**	0.2085810
	forest	white-yellow	1	0.2121157	3.201303	0.135	0.0550039
		white-orange	1	1.4004919	23.905818	**<0.01**	0.2601121
		yellow-orange	1	0.6356507	10.118621	**<0.01**	0.1188767
Avian	d65	white-yellow	1	0.1576632	2.150818	0.351	0.0376341
		white-orange	1	0.9088843	14.247268	**<0.01**	0.1732248
		yellow-orange	1	0.3473225	5.215843	**0.018**	0.0650226
	forest	white-yellow	1	0.09929183	1.285365	0.729	0.0228366
		white-orange	1	0.3494738	5.077892	**0.018**	0.069486
		yellow-orange	1	0.1506068	2.120343	0.294	0.027494
Snake	d65	white-yellow	1	0.1436898	2.030032	0.372	0.03559583
		white-orange	1	0.9895660	15.628953	**<0.01**	0.18688448
		yellow-orange	1	0.4439913	6.746494	**<0.01**	0.08252946
	forest	white-yellow	1	0.08539601	1.136560	0.927	0.0202463
		white-orange	1	0.4311498	6.274411	**<0.01**	0.0844761
		yellow-orange	1	0.18919941	2.659063	0.192	0.03424022

## Discussion

Our results indicate that *Podarcis erhardii* and some of their most important visual predators can distinguish between throat morph colors across most illuminance contexts. These results provide support for the notion that *P. erhardii* can perceive variation in conspecific ventral coloration as discrete categories, which is a prerequisite for morph-specific sexual selection. However, raptors, and in some instances colubrid snakes, cannot discern achromatic components of the throat signal between morphs, whereas wall lizards can (Table 3). Furthermore, through the eyes of a lizard, orange morphs are more noticeably different than white and yellow morphs across two different lighting environments (Fig 3). These results suggest that chromatically distinct *P. erhardii* color morphs may employ achromatic visual signals in lighting contexts that minimize the risk of predators intercepting their chromatic signals.

Research suggests that the pigment-based (i.e., carotenoids, pteridine) and structurally-based (i.e., blue, violet, UV) coloration found in numerous lizard species can function as signals of mate quality for males [15,16,53] and females alike [66]. The production of costly color-producing compounds can impose a trade-off for resource allocation that limits other important physiological processes [67], thus producing an honest indication of fitness. If polymorphic throat coloration in this species signals morph-specific information (e.g., size, aggression, health status; [37,38]), then the visual system of this species must be capable of distinguishing between morph coloration. Our findings here on *P. erhardii* align with several other visual modelling studies on other *Podarcis* species that demonstrate visual discrimination between morphs [14,53]. Colorimetric variables, such as hue, brightness, saturation, and UV chroma are correlated to other ecological and fitness metrics in color polymorphic lizards [67], including parasite load [68], immune response [69, but see 70], fighting ability [17–19] and territory size [71]. Previous research in *P. erhardii* found that there are no significant differences in these colorimetric variables between sexes of the same color morph but there are substantial differences between color morphs [37]. More research on color morph mate choice and morph-specific mating behavior is needed to determine if this species is using polymorphic throat colors in reproductive strategies and mating decisions.

Interestingly, we observed a divergent UV-spectra spike centered around 370 nm for a subset of the white morph lizards (males, *n* = 3; females, *n* = 7; Fig 1), and a smaller subset of the orange morph females (*n* = 4). In the related Tyrrhenian wall lizard, *P*. *tiliguerta*, ventral scale UV-blue patches may signal information about individual resource-holding potential [53]; however, these results were restricted to only male lizards and UV-blue patches present on the flanks of the lizards, not white throat color patches. Previous research demonstrates sexual dichromatism of UV coloration in other lizard species [72,73], but largely, the focus on UV coloration research is centered on sexual selection for male lizards and indicators of mate quality. As far as we know, this is the first study to document this divergent UV-coloration reflectance spike in polymorphic female lizards. It is often assumed that females are more drab and less bright than males because they seem so to our visual system, but this may not be true through the eyes of a wall lizard [37]. Further investigation should be conducted into whether throat UV reflectance is a regularly occurring feature of white and orange morphs in *Podarcis*; and if so, what factors govern its presence, what social behaviors and interactions is it associated with, and what information does it communicate, if any?

An associated cost of distinct coloration is an increased visibility to predators [20–22], who often have visual systems attuned to ‘eavesdrop’ on the communication signals of their prey [74]. Indeed, our visual models suggest that birds and snakes can perceive the difference between three shades of *P. erhardii* throat coloration as discrete colors, even under sub-optimal illuminance conditions (Fig 3). However, it is important here to acknowledge that throat color badges are likely not often visible from an overhead avian perspective. Signal partitioning [74–76] is a means through which animals can diminish their conspicuousness to predators through behavioral changes or the obfuscation of ornaments from a typical predatory viewing angle. Throughout its range, *P. erhardii* is predated upon by colubrid snakes [45]. From the perspective of these visual predators that can also sense UV [31], throat color badges are likely visible on a more regular basis due to their angle of observation from the ground. Furthermore, the relative conspicuousness of a given throat color is impacted by its contrast against a natural background. *Podarcis* lizards often utilize rocky substrates like granite and schist, as well as vegetated substrates like moss and grass [15]; across these variable contexts, predators perceive the relative conspicuousness of *Podarcis* coloration differently. Future research should assess whether throat coloration in *P. erhardii* is duller or otherwise differs in terms of colorimetric variables between populations where snake predators are present versus absent.

Microhabitat preferences among sympatric *P. erhardii* color morphs differ, which can impact how coloration is perceived and distinguished by conspecifics and predators alike. In this species, orange morphs were found utilizing vegetative cover and in shade significantly more often than white and yellow morphs that were found in more open, warmer, and well-lit microhabitat [44]. Orange morph microhabitat use was associated with an increased ectoparasitic burden for orange morphs [44]. In principle, utilizing this vegetative cover should offer some additional benefit to justify its ecological cost. Color conspicuousness (i.e., chromatic distance) is impacted by variable ambient light conditions—such as those of low-light, shaded vegetation as compared to bright, open stone wall refugia—since the availability of certain light wavelengths is restricted [61]. As light levels decrease, the signal-to-noise ratio decreases to a point where color vision may become unreliable [77,78]; under these conditions, the achromatic (i.e., intensity or luminance) information channel may instead be relied upon. We found that for the lizard visual system, the mean achromatic distance between all morph pairs exceeded 3 JND under both lighting contexts, with the exception of the white-yellow morph pair in forest shade (Fig 3). However, for the raptor visual model, no morph pair surpassed 1 JND in mean achromatic contrast, whereas our snake visual model only exceeded 3 JND for the orange-white pair and the orange-yellow pair in standard daylight. We did not find other instances in the literature where conspecifics could perceive achromatic differences that their predators could not. However, past research on violet-sensitive avian predators has emphasized the importance of chromatic contrast of prey against natural backgrounds for hunting [13,79]. In terms of object detection and identification, achromatic contrasts are considered less reliable because light intensity can be highly variable in foliage microhabitat [24,79]. *P. erhardii* orange morphs are the most chromatically distinct (Table 1; [see 37]) and may run an increased risk of predation [21]. However, if achromatic contrast is indeed a viable means of conveying fitness information, by occupying highly vegetated microhabitat, orange morphs can reduce the likelihood of a predator noticing their bold chromatic coloration while broadcasting achromatic fitness information to conspecifics. Color morphs also choose different substrate types, with orange morphs often being found on darker substrates such as dirt, rock, and plant matter, white morphs on artificial and stone wall substrates in well lit areas, and yellow morphs exhibiting intermediate perching preferences (K.M. Brock, unpublished data). Further research on the conspicuousness of these color morphs compared to their perching backgrounds and through the eyes of different predators under variable lighting conditions is necessary.

This study indicates that the visual systems of *Podarcis erhardii,* as well as bird and snake predators, can chromatically distinguish between the three throat color morphs of this lizard in standard daylight and forest shade illuminance conditions. Although lizards also retained the ability to discriminate between achromatic morph colors, raptors and snakes did not, which may offer insight into previously observed differences in microhabitat usage between *P. erhardii* conspecifics [44]. These results broadly corroborate previous research into the visual systems of lizards and those of their predators in the *Podarcis* genus and their respective abilities to recognize variation in morph coloration as discrete colors [14,47,53]. Understanding the conspicuousness of morph coloration to conspecifics and predators can help explain the mechanisms that generate, maintain, and erode morph diversity within and among populations of *P. erhardii*. Future research that determines relative morph-specific predation risk by visual predators will shed further insights on color morph maintenance in this system.

## Supporting information

S1 Data R code and study data including lizard throat chroma. (ZIP)
